# Development of TaqMan probes targeting the four major celiac disease epitopes found in α-gliadin sequences of spelt (*Triticum aestivum* ssp. *spelta*) and bread wheat (*Triticum aestivum* ssp. *aestivum*)

**DOI:** 10.1186/s13007-017-0222-2

**Published:** 2017-09-06

**Authors:** Benjamin Dubois, Pierre Bertin, Yordan Muhovski, Emmanuelle Escarnot, Dominique Mingeot

**Affiliations:** 1 Département Sciences du vivant, Centre wallon de Recherches agronomiques (CRA-W), Chaussée de Charleroi, 234, 5030 Gembloux, Belgium; 2 Département Sciences du vivant, Centre wallon de Recherches agronomiques (CRA-W), Rue de Liroux, 4, 5030 Gembloux, Belgium; 3 Earth and Life Institute – Agronomy, Université catholique de Louvain (UCL), Croix du Sud, 2 bte L7.05.11, 1348 Louvain-la-Neuve, Belgium

**Keywords:** Spelt, α-gliadin, Celiac disease, TaqMan probe, Reference genes, Gluten

## Abstract

**Background:**

Celiac disease (CD) is caused by specific sequences of gluten proteins found in cereals such as bread wheat (*Triticum aestivum* ssp. *aestivum*) and spelt (*T. aestivum* ssp. *spelta*). Among them, the α-gliadins display the highest immunogenicity, with four T-cell stimulatory epitopes. The toxicity of each epitope sequence can be reduced or even suppressed according to the allelic form of each sequence. One way to address the CD problem would be to make use of this allelic variability in breeding programs to develop safe varieties, but tools to track the presence of toxic epitopes are required. The objective of this study was to develop a tool to accurately detect and quantify the immunogenic content of expressed α-gliadins of spelt and bread wheat.

**Results:**

Four TaqMan probes that only hybridize to the canonical—i.e. toxic—form of each of the four epitopes were developed and their specificity was demonstrated. Six TaqMan probes targeting stable reference genes were also developed and constitute a tool to normalize qPCR data. The probes were used to measure the epitope expression levels of 11 contrasted spelt accessions and three ancestral diploid accessions of bread wheat and spelt. A high expression variability was highlighted among epitopes and among accessions, especially in Asian spelts, which showed lower epitope expression levels than the other spelts. Some discrepancies were identified between the canonical epitope expression level and the global amount of expressed α-gliadins, which makes the designed TaqMan probes a useful tool to quantify the immunogenic potential independently of the global amount of expressed α-gliadins.

**Conclusions:**

The results obtained in this study provide useful tools to study the immunogenic potential of expressed α-gliadin sequences from Triticeae accessions such as spelt and bread wheat. The application of the designed probes to contrasted spelt accessions revealed a high variability and interesting low canonical epitope expression levels in the Asian spelt accessions studied.

**Electronic supplementary material:**

The online version of this article (doi:10.1186/s13007-017-0222-2) contains supplementary material, which is available to authorized users.

## Background

Gluten is a group of water-insoluble proteins which display useful properties for dough formation. These proteins are found in the seed of cereals such as bread wheat (*Triticum aestivum* L. ssp. *aestivum*), spelt [*T. aestivum* ssp. *spelta* (L.) Thell.], barley (*Hordeum vulgare* L.) and rye (*Secale cereale* L.). The gluten complex is composed of monomeric gliadins and polymeric glutenins which confer visco-elasticity properties to the dough [1, 2].

The consumption of gluten-containing foods can lead to several disorders in a fraction of the population, the best known being mediated by the adaptive immune system: celiac disease (CD) and wheat allergy (WA). They are both mediated by a T-cell activation in the gastrointestinal mucosa. WA involves immunoglobulins (Ig)-E that link to repeat sequences in wheat proteins whereas CD is an autoimmune disorder with a genetic predisposition of the patient, mediated by Ig-A and Ig-G antibodies. Beside these two disorders, a novel pathologic entity—called non-celiac gluten sensitivity (NCGS)—has gained importance and involves neither autoimmune nor allergic mechanisms. People affected by this illness display symptoms similar to CD but with usually normal small intestinal histology. These patients become healthier when gluten or wheat is withdrawn from the diet [3–6]. CD affects about 1% of the human population and is thus one of the most important food sensitivities worldwide [6, 7]. Some gluten peptides are partially resistant to proteolysis and are presented by HLA-DQ2 or -DQ8 molecules on antigen-presenting cells (APC) to T-cells which are hence activated. The subsequent production of inflammatory cytokines leads to small intestinal injuries such as reduction of villus height and a decrease of its absorptive capacity, which may cause severe malnutrition [8, 9]. Intra- and extraintestinal symptoms are encountered like diarrhea, bowel pain, fatigue, weight loss, anemia, osteoporosis, headaches and growth retardation [5, 6, 10, 11].

Different strategies are currently being investigated to address the CD problem, such as gluten detoxification, modulation of mucosal permeability, antigen presentation blockade, raising monoclonal antibodies against inflammatory cytokines, inhibition of T-cell recruitment, or oral tolerance induction [10]. Among them, the development of new cereal varieties lacking immunogenic gluten peptides but still displaying good baking properties is a promising approach given the high variability existing in bread wheat and its related taxa [10, 12]. Spelt is one of these taxa and is a member of the *T. aestivum* species, just like bread wheat. It is particularly interesting because of the high genetic diversity held in spelt germplasm collections [13–15]. Moreover, genetic variations in their immunogenic potential, their bread-making qualities and their content in proteins, lipids, micronutrients and fibers have been pointed out [13, 16–19].

Gliadins are usually classified into the α/β-, γ- and ω-types. The α-gliadins are the most studied ones since they trigger the strongest T-cell activation [8, 20–22]. The Gli-2 loci, which encode α-gliadins, are located on the short arm of the three homeologous chromosomes from group 6 and they include a number of α-gliadin gene copies that might reach 150 per haploid genome in some accessions [23]. However, a high proportion of these copies are pseudogenes: Ozuna et al. [24] showed that 39, 76 and 63% of α-gliadin genes from diploid, tetraploid and hexaploid wheat species respectively displayed a premature stop codon. The α-gliadin strong immunogenicity is mainly due to four T-cell stimulatory epitopes. Two are major epitopes (the overlapping DQ2.5-glia-α1 and -α2 epitopes) and the other two are minor epitopes (the DQ2.5-glia-α3 and DQ8-glia-α1 epitopes). The DQ2.5-glia-α2 epitope can be displayed in one, two or three copies and leads, when three copies are present, to the most immunogenic fragment of α-gliadin sequences known as the 33-mer fragment [25, 26]. Each of these four epitopes can be displayed in its canonical form, which is toxic, but some amino acid substitutions or deletions can reduce or suppress antigenic properties, according to the allelic variant [27]. Interestingly, the epitope variants highlighted in bread wheat α-gliadin sequences by Mitea et al. [27] were similar to those found in spelt accessions [16].The multigenic character of the α-gliadin family results in a very high allelic variability and leads, among other things, to high variations in the immunogenic content from one α-gliadin sequence to another. The exploitation of this variability to develop safe spelt or wheat cultivars will probably require the combination of classical breeding and molecular technologies [12], and tools to infer the presence of sequences coding for toxic epitopes are needed.

ELISA test kits have been developed to measure the amount of gluten in food samples and to detect gluten contamination. Generally speaking, they are based on different antibodies [28–33] which have been raised against gluten proteins. However, although ELISA kits provide information on toxicity, the restricted specificity of the antibodies limits the accuracy of ELISA tests regarding the immunogenic potential of varieties. Indeed, each antibody is raised against only a short stretch of one of the toxic epitopes, which may lead to the simultaneous detection of non-toxic epitope variants together with the canonical—toxic—epitope form, because the mutation is located outside the antibody hybridization site. It has been shown that G12 and A1 antibodies display the highest affinity for canonical epitopes included in the 33-mer fragment, but they also recognize allelic variants of these epitopes to a lesser extent [30]. Also, no ELISA kit can be used to study the four major α-gliadin T-cell stimulatory epitopes at the same time. Furthermore, antibodies used in ELISA test kits generally recognize more than one site. For example, the R5 antibody links to the epitope QQPFP and the related sequences QQQFP, LQPFP and QLPFP [33], but only one of these (LQPFP) is found in the composition of the four major canonical T-cell stimulatory epitopes of α-gliadin sequences. Lastly, such antibodies generally recognize different types of gluten proteins, such as the Skerritt antibody that detects ω-gliadins and HMW glutenin subunits. In addition to ELISA kits, some other techniques have recently been developed to detect and quantify different CD-immunogenic peptides, such as aptamer receptors and liquid chromatography combined with mass spectrometry [34–36].

The multigenic nature of α-gliadins and the high proportion of pseudogenes would require markers able to detect and quantify toxic sequences in expressed genes. The TaqMan probe system is one of the most commonly used to perform a quantitative detection of amplicons [37], and is particularly adapted to the study of the epitope expression profile since it makes it possible to highlight single-nucleotide polymorphisms (SNPs) and thus to discriminate allelic variants [38, 39].

The objective of this study was to develop and validate a tool to measure the immunogenic content for CD patients held in expressed α-gliadins. This objective was pursued by (1) developing epitope-specific TaqMan probes that only hybridize to the canonical form of the four epitopes, (2) selecting stable reference genes and developing specific TaqMan probes to normalize qPCR data and (3) validating the designed probes through their application to cDNA samples from 11 contrasted spelt accessions and three diploid species representative of the ancestral genomes of spelt and bread wheat.

## Methods

### Plant materials

Eleven contrasted spelt accessions selected in a previous genetic diversity study [16] as a representative panel of spelt diversity were used (BEL08, DK01, SPA03, BUL04, GER11, GER12, TAD06, SWI23, US06, Iran77d and IRA03). Three diploid accessions representative of the three ancestral genomes of spelt and bread wheat were added to this selection (Table 1): LB01 (*Triticum urartu* Tumanian ex Gandilyan, A genome), TR08 (*Aegilops speltoides* Tausch, S genome which is suspected to be the ancestor of the B genome) and TR10 (*Aegilops tauschii* Cosson, D genome). The accessions were kindly provided by the United States Department of Agriculture (USDA, Washington, USA), the Vavilov Institute of Plant Genetic Resources (VIR, Saint Petersburg, Russia) and the Center for Genetic Resources (CGN, Wageningen, The Netherlands). They were grown in 2013–2014 in Gembloux (Belgium) in field conditions. To ensure the self-pollination of the ears, they were enclosed with cellophane bags. All the immature grains were harvested 20 days post-anthesis (DPA), immediately frozen in liquid nitrogen and stored at −80 °C.

**Table 1:** Genetic material used to analyze the α-gliadin canonical epitope expression with TaqMan probes

Species	Genome	Name	Accession name	Accession number	Germplasm	Provenance country
*Triticum aestivum* ssp. *spelta*	ABD	BEL08	69Z6,485	PI348315	USDA (Washington, USA)	Belgium
		DK01	DN-2267	PI361811	USDA (Washington, USA)	Denmark
		SPA03	69Z6,752	PI348572	USDA (Washington, USA)	Spain
		BUL04	Ungarn	PI295063	USDA (Washington, USA)	Bulgaria
		GER11	69Z6,275	PI348114	USDA (Washington, USA)	Germany
		GER12	69Z6,282	PI348120	USDA (Washington, USA)	Germany
		TAD06	–	K 52437	VIR (Saint Petersburg, Russia)	Tajikistan
		SWI23	69Z6,93	PI347939	USDA (Washington, USA)	Switzerland
		US06	69Z5,73	PI355595	USDA (Washington, USA)	USA
		Iran77d	Iran 77d	CGN 06533	CGN (Wageningen, The Netherlands)	Iran
		IRA03	Iran 416A	CGN12270	CGN (Wageningen, The Netherlands)	Iran
*Triticum urartu*	A	LB01	G3178	PI 428293	USDA (Washington, USA)	Lebanon
*Aegilops speltoides*	S*	TR08	2733	PI 170204	USDA (Washington, USA)	Turkey
*Aegilops tauschii*	D	TR10	79TK057-322-1	PI 486267	USDA (Washington, USA)	Turkey

The 11 spelt accessions are contrasted accessions previously selected after a genetic diversity analysis [14]. *T. urartu*, *Ae. speltoides* and *Ae. tauschii* are diploid species representative of the three ancestral genomes (A, B and D, respectively) of spelt and bread wheat. They were selected to validate the specificity of the designed TaqMan probes

*USDA* United States Department of Agriculture, *VIR* Vavilov Institute of Plant Genetic Resources, *CGN* Center for Genetic Resources

* The B genome is hypothesized to be an altered S genome; *Ae. sepltoides* is therefore taken as the closest representative of the B genome

### RNA extraction, cDNA synthesis and plasmid extraction

Total RNA was extracted for each accession from 100 mg of ground seeds using the NucleoSpin^®^ RNA Plant kit (Macherey-Nagel, Germany) and quantified by spectrometry. First-strand cDNA was synthesized with oligo(dT)_18_ primer from 250 ng RNA using the RevertAid H Minus First Strand cDNA Synthesis Kit (Thermo Scientific) in a volume of 20 µl. It was then quantified by spectrometry.

The plasmids of previously cloned α-gliadin sequences [16] were extracted with the GeneJet Plasmid Miniprep Kit (Thermo Scientific) after the overnight incubation of bacterial cultures at 37 °C. The GeneBank accession numbers of the sequences are reported in Additional file 1.

### Design of epitope-targeting primers and probes

For each epitope, primers were developed (Table 2) so that they hybridize in conserved regions on both sides of the epitope. The design was carried out by aligning previously cloned spelt α-gliadin sequences [16] with the Vector NTI software (v6.0).

**Table 2:** List of the primers and TaqMan probes designed in this study

Target epitope/gene	Oligonucleotide	Sequence
*Epitopes*		
DQ2.5-glia-α1	Forward primer	5′-GCAACCATTTCCATCACAACWAC-3′
	Reverse primer	5′-GTGSTTGCGAATACTGTGGTTG-3′
	Probe	5′-FAM-WTCCRCAGCCGCAACTACCA-TAMRA-3′
DQ2.5-glia-α2	Forward primer	5′-GCAACCATTTCCATCACAACWAC-3′
	Reverse primer	5′-GTGSTTGCGAATACTGTGGTTG-3′
	Probe	5′-FAM-AGCCGCAACTACCATATCCGC-TAMRA-3′
DQ2.5-glia-α3	Forward primer	5′-GCAACCATTTCCATCACAACWAC-3′
	Reverse primer	5′-GTGSTTGCGAATACTGTGGTTG-3′
	Probe	5′-FAM-TTCGACCACAACAACCATATCCAC-TAMRA-3′
DQ8-glia-α1	Forward primer	5′-CCACAATGTYGTTCATGCTATTATTCTGC-3′
	Reverse primer	5′-CAGAGCCCTGGGCCTGTGG-3′
	Probe	5′-FAM-AGGGCTCCTTCCAGCCAT-TAMRA-3′
*Reference genes*		
ADP-ribosylation factor (ARF)	Forward primer	5′-GCTCTCCAACAACATTGCCAAC-3′
	Reverse primer	5′-GCTTCTGCCTGTCACATACGC-3′
	Probe	5′-FAM-CAAGAAACAAACGTGCTGGATGTC-TAMRA-3′
Similar to RNase L inhibitor-like protein (RLI)	Forward primer	5′-CGATTCAGAGCAGCGTATTGTTGC-3′
	Reverse primer	5′-AGTTGGTCGGGTCTCTTCTAAATGTAATG-3′
	Probe	5′-FAM-CTTAGCGGACAAGGTTATTGTTTATGAGG-TAMRA-3′
Vacuolar ATP synthase 16 kDa proteolipid sub. (VAS)	Forward primer	5′-GCTGGAGTGCTCGGTATCTACGG-3′
	Reverse primer	5′-TGCGAAGATGAGGATGAGGATCA-3′
	Probe	5′-FAM-ATCGGCATTGTTGGTGATGCT-TAMRA-3′
Ubiquinol-cytochrome C reduct. iron-sulfur sub. (UCC)	Forward primer	5′-CCTGCCCCGTACAACCTTGAG-3′
	Reverse primer	5′-TCACCGTTGCGATAGTCCTGAAAC-3′
	Probe	5′-FAM-ACAGGAGTGAATTCCTGTTGCGC-TAMRA-3′
GABA-receptor-associated protein (GABA)	Forward primer	5′-TTACGAGGAGAACAAGGACGAGGA-3′
	Reverse primer	5′-CAGGAGGCATTCAGAGCGATTG-3′
	Probe	5′-FAM-CACCTTCGGATTGCTCTAGATGGC-TAMRA-3′
Protein of unknown function [DUF52 family] (DUF52)	Forward primer	5′-TGGTGCCATTCACAAATCAATCG-3′
	Reverse primer	5′-GCGAACAAACCCGACCTTAATCTTC-3′
	Probe	5′-FAM-CATGGAGATCATAGAGACTGGTGACC-TAMRA-3′
Cell division control protein (CDC)	Forward primer	5′-CAAATACGCCATCAGGGAGAACATC-3′
	Reverse primer	5′-CGCTGCCGAAACCACGAGAC-3′
Protein transport protein Sec23A (SEC23)	Forward primer	5′-AGCAATTCGCACAATTATTACAAGCTC-3′
	Reverse primer	5′-GATGCTCACAGAAGACCTGGAAGC-3′
Superoxide dismutase [Cu–Zn] (SOD)	Forward primer	5′-CCTTACTGGACCAAATTCAATTGTTGG-3′
	Reverse primer	5′-GGTGCACACTAACAAGTGATCAAAGATC-3′
S-adenosylmethionine decarboxylase (SAD)	Forward primer	5′-GGCTGGACAAGAAGAAGGCCTCT-3′
	Reverse primer	5′-ATGGATGGTGGAGACGGCAGAT-3′

The primers and probes were designed to measure the expression levels of the four α-gliadin major T-cell stimulatory epitopes in their canonical form and the expression levels of reference genes. Each TaqMan probe was labeled with the fluorogenic dye FAM (fluorescein) at its 5′ end and with the quencher TAMRA (tetramethylrhodamine) at its 3′ end

The probe development was carried out in accordance with the following important probe characteristics: (1) the amplicon should have a length of between 75 and 150 bp; (2) no guanine residue should be located at the 5′ end of the probe as it quenches the fluorescent dye; (3) runs of identical nucleotides should be avoided, especially four or more consecutive guanine residues; (4) the probe should have a Tm of 65–67 °C and a minimum length of 13 nucleotides; (5) the SNP position in the probe should be as central as possible to maximize the detection specificity [40]. TaqMan probes focusing on the four major T-cell stimulatory epitopes DQ2.5-glia-α1, DQ2.5-glia-α2, DQ2.5-glia-α3 and DQ8-glia-α1 were designed so that they only hybridized to the canonical form of each epitope (Table 2). This was done by aligning spelt α-gliadin sequences cloned in a previous study [16] with the Vector NTI software. The probes were designed to cover the entire regions where epitope mutations are found in spelt and bread wheat sequences.

### Quantitative PCR

Amplifications with TaqMan probes were carried out using 10 µl of Takyon™ No ROX Probe 2× Mastermix dTTP Blue (Eurogentec, Belgium), 300 nM of each primer and 100 nM of TaqMan probe labeled with the FAM fluorophore and the TAMRA quencher (Eurofins Genomics, Germany), 400 ng of cDNA and nuclease-free water (Thermo Scientific) for a total volume of 20 µl. For amplifications using the SYBR^®^ dye, each reaction was carried out using 10 µl of Takyon™ No ROX SYBR^®^ 2x MasterMix dTTP Blue (Eurogentec, Belgium), 300 nM of each primer, 400 ng of cDNA and nuclease-free water for a total volume of 20 µl. Samples were loaded in a Hard-Shell^®^ 96-Well PCR skirted white plate and sealed with a Microseal^®^ ‘B’ PCR Plate Sealing Film (Bio-Rad). PCR amplifications were performed by the C1000 Touch™ Thermal Cycler coupled to the CFX96™ Real-Time detection system (Bio-Rad). The following thermal cycling protocol was used: initial denaturation at 95 °C for 3 min followed by 40 cycles at 95 °C for 10 s and 69 °C for 1 min.

### Validation of primer efficiency and TaqMan probe specificity

Each set of primers was validated for its efficiency by carrying out qPCR amplifications with successive tenfold cDNA dilutions. For each qPCR reaction, the C_t_ (threshold cycle) value, defined as the fractional qPCR cycle number at which the fluorescent signal crosses an arbitrarily placed threshold, was measured for quantification. A calibration curve plotting the logarithm of the cDNA concentration on the x-axis and the corresponding C_t_ values on the y-axis was drawn. The efficiency was then calculated based on the curve slope: Efficiency (%) = [(10^−1/slope^) − 1] * 100. The primers were selected so that their efficiency was comprised between 96 and 100%.

The four probes were checked for their specificity by performing qPCR amplifications with previously cloned α-gliadin sequences [16] displaying either the canonical form or one of the allelic variants of the four epitopes (see Additional file 1). The probe specificity was validated when a fluorescent signal was measured only for the α-gliadin clone showing the canonical epitope.

### Design of primers and probes targeting reference genes

With the aim of accurately normalizing qPCR data, Paolacci et al. [41] evaluated the expression stability of 32 reference genes in different wheat tissues: roots, shoots, stems, flag leaves, spikes, single floral organs and caryopses. On this basis, ten of the most stable genes were selected for the present study, and primers and TaqMan probes were designed to quantify their expression. Primers and probes were developed based on wheat contig sequences from the TIGR wheat Gene index database (ftp://occams.dfci.harvard.edu/pub/bio/tgi/data/Triticum_aestivum/, Table 2).

For each reference gene, TIGR contigs mentioned in Paolacci et al. [41] were aligned with Vector NTI and primers were designed in conserved regions to amplify 150–200 bp-long amplicons. The amplifications were checked in qPCR experiments with SYBR^®^ dye on 400 ng of cDNA from 11 spelt accessions and three diploid species representative of the A, B and D genomes to ensure that the reference genes are present in the three genomes. Each cDNA sample was loaded in triplicate. The specificity of the primers targeting reference genes was checked by running the qPCR products on a 2% agarose gel.

TaqMan probes were then designed for the reference genes that showed an amplification, and each set of primers was validated for its efficiency (same procedure as detailed above). These probes were tested on the 14 accessions. The geNorm software was used following the procedure detailed by Vandesompele et al. [42] to calculate the M and V values. The M value can be used to classify the reference genes according to their expression stability by the stepwise exclusion in the calculation process of the least stable gene. The V value helps to determine the appropriate number of reference genes to use by analyzing the expression stability of the two most stable genes and by the stepwise addition of the next most stable reference gene to the analysis.

### Epitope expression profiling with designed TaqMan probes

The four TaqMan probes designed to focus on the canonical epitopes and the four TaqMan probes targeting the four most stable reference genes were applied to the cDNA of the 11 spelt and three diploid accessions. Each accession was tested in two biological replications and each sample was loaded in triplicate. Measured C_t_ values were reported to C_t_ values of a calibrator. For the epitope-targeting probes, the calibrator was a previously cloned α-gliadin sequence (GER12_63, accession number KX174081) displaying one copy of each of the four canonical epitopes. This enabled the comparison of expression values from one epitope to another. For each reference gene, the calibrator consisted of the sample with the lowest C_t_ value, according to the geNorm user manual instructions. To allow comparison from one accession to another, the epitope expression levels were normalized to the expression of the reference genes, thanks to the calculation of normalization factors. The calculation of the epitope relative quantities was carried out using the $2^{{\Delta {\text{C}}_{\text{t}} }}$ method, where ΔC_t_ corresponds to C_t, calibrator_ − C_t, sample_, and dividing by the normalization factor as recommended by Vandesompele et al. [42].

### Alpha-gliadin expression profiling

The global α-gliadin expression level was evaluated in each accession by SYBR^®^ qPCR amplifications using primers hybridizing on both sides of the DQ2.5-glia-α1, -α2 and -α3 epitopes (Table 2). Melting curve analyses followed qPCR amplifications to check that only one amplicon was amplified in each sample: the temperature was increased from 65 to 95 °C, with increments of 0.5 °C for 5 s (Additional file 2). Primers designed in TaqMan experiments to target reference genes were also used in qPCR amplification with SYBR^®^ dye for normalization. The sample with the lowest C_t_ value was chosen as the calibrator for the $2^{{\Delta {\text{C}}_{\text{t}} }}$ calculation.

## Results

### Development of TaqMan probes specific to the four canonical α-gliadin epitopes

**Fig. 1:**
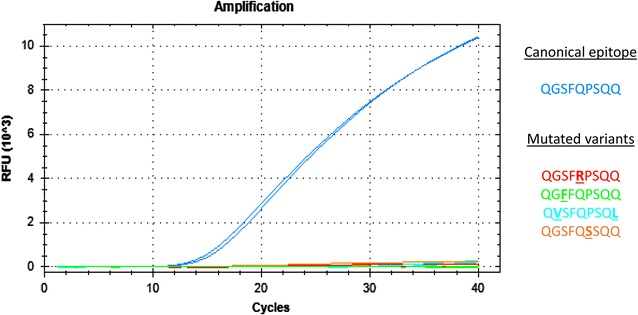
Illustration of the specificity of the TaqMan probe designed to target the canonical DQ8-glia-α1 epitope. The high fluorescent signal observed with the α-gliadin clone displaying the canonical epitope (QGSFQPSQQ) combined with the insignificant or absent fluorescence with the clones containing the allelic variants validated the probe specificity. *RFU* Relative fluorescent units

Four TaqMan probes and primer pairs were designed to focus on the canonical form of the four major α-gliadin T-cell stimulatory epitopes recognized by the immune system of CD patients: DQ2.5-glia-α1, DQ2.5-glia-α2, DQ2.5-glia-α3 and DQ8-glia-α1 epitopes (Table 2). These epitopes are found in α-gliadin sequences from both spelt and bread wheat, as previously stated. In order to avoid any overestimation of the immunogenic content, the probes could not hybridize to any known allelic variant of these epitopes. The specificity of each probe was confirmed by qPCR analyses: a fluorescent signal was clearly observable with the α-gliadin clones displaying the canonical epitope, while it was insignificant or absent with clones containing allelic variants (Fig. 1). The specificity of the probes was further confirmed on the cDNA of the three diploid species representative of the ancestral genomes (A, B and D) of spelt and bread wheat (see below). The primer efficiency was comprised between 96.4 and 99.5%, according to the primer pairs (see Additional file 3).

### Selection of stable reference genes and development of their specific primers and TaqMan probes

**Fig. 2:**
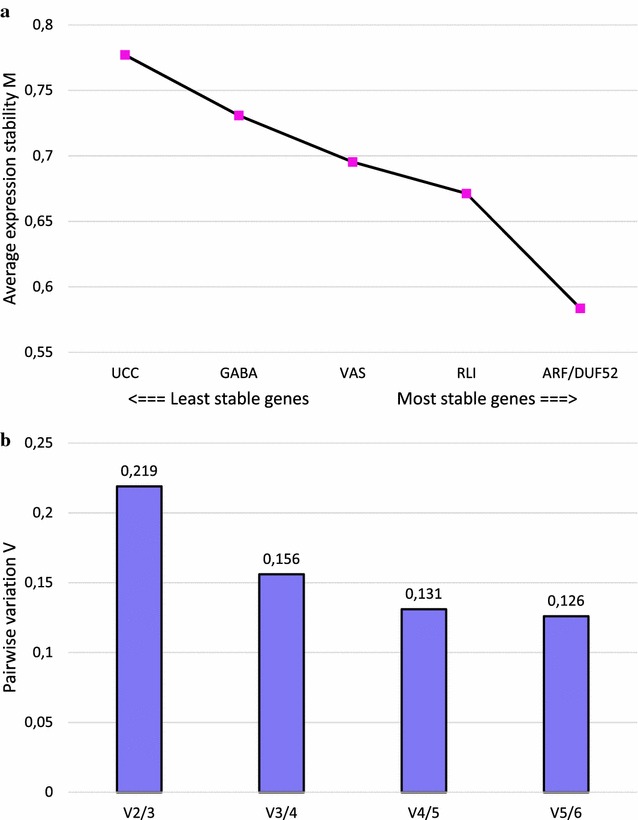
geNorm output charts of M and V values for the six tested reference genes. **a** Average expression stability M of the remaining reference genes after stepwise exclusion of the least stable gene. **b** Pairwise variation V between two sequential normalization factors containing an increased number of reference genes, calculated for the determination of the optimal number of reference genes for normalization

In order to normalize qPCR data, Paolacci et al. [41] evaluated the expression stability of 32 reference genes in different wheat samples. Based on their results, ten of the most stable genes were selected for the present study. Sets of primers and probes were developed to analyze their expression stability in immature seeds harvested 20 DPA from spelt and diploid species representative of the A, B and D genomes (Table 2). Among them, four reference genes (CDC, SOD, SAD and Sec23) were discarded as they showed under- or no expression in one of the three diploid species. Among the six remaining reference genes tested, the analysis with the geNorm software showed that ARF and DUF52 were the two most stable according to their average expression stability (M value, Fig. 2a), followed by RLI, VAS, GABA and UCC.

The evolution of the pairwise variation (V) between two sequential normalization factors (NF_n_ and NF_n+1_) showed decreasing values when an additional reference gene was used for calculation (Fig. 2b). The combination of the four most stable reference genes resulted in a V value of 0.156 between NF_3_ and NF_4_, and was chosen as the best compromise between an optimal cut-off (0.15) below which the inclusion of an additional reference gene is not required, and technical adequacy. The reference genes ARF, DUF52, RLI and VAS were thus selected to normalize qPCR data. The primers targeting these reference genes showed good efficiency (comprised between 97 and 100%, see Additional file 3) and specificity (Additional file 4).

### Epitope expression profile

**Fig. 3:**
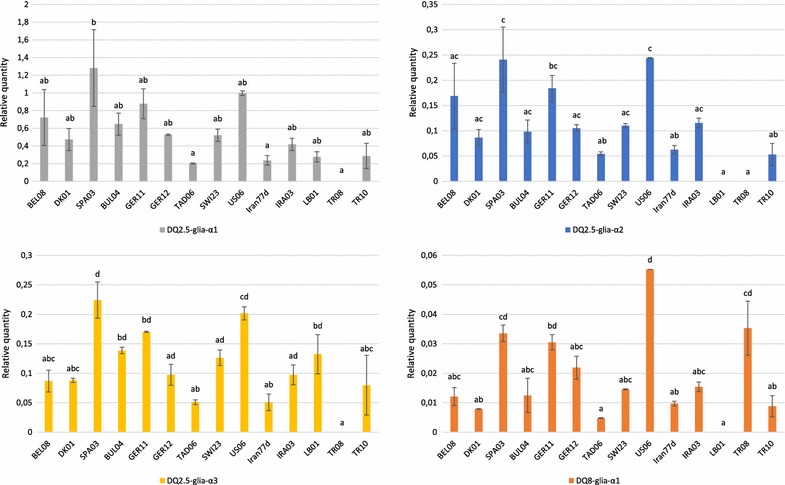
Relative abundance of the four canonical α-gliadin epitopes among contrasted spelt accessions and diploid species. Developed TaqMan probes were used for the relative quantification of the expression of the four major α-gliadin T-cell stimulatory epitopes in their canonical form among 11 contrasted spelt accessions and the three diploid accessions representative of the ancestral genomes of spelt and bread wheat: *T. urartu* (LB01, A genome), *Ae. speltoides* (TR08, B genome) and *Ae. tauschii* (TR10, D genome). The relative quantities were calculated by dividing the $2^{{\Delta {\text{C}}_{\text{t}} }}$ values by a normalization factor obtained through the expression analysis of four stable reference genes. Data are presented with standard error of the mean and significant differences detected by Tukey’s multiple comparison test are shown by *different letters*

The qPCR experiments were carried out on cDNA samples of 11 spelt and three diploid accessions to measure Ct values (Additional file 5). These values were then used to calculate the relative quantity of the four canonical T-cell stimulatory epitopes compared to reference genes (Fig. 3). From one accession to another, high variations in the expression levels were displayed but the same trend was observed for each epitope. The analysis of variance showed, from one accession to another, statistically significant differences in the expression levels of the DQ2.5-glia-α1 (ANOVA F_13_ = 4.3829; *P* = 4.874e−3), DQ2.5-glia-α2 (ANOVA F_13_ = 7.9597; *P* = 2.202e−4), DQ2.5-glia-α3 (ANOVA F_13_ = 8.4628; *P* = 1.558e−4) and DQ8-glia-α1 (ANOVA F_13_ = 12.971; *P* = 2.251e−5) epitopes. The spelt accessions SPA03 and US06 showed the highest expression values for the DQ2.5-glia-α1, DQ2.5-glia-α2 and DQ2.5-glia-α3 epitopes, and US06 displayed the highest expression for the DQ8-glia-α1 epitope. Conversely, the Tajik TAD06 and Iranian Iran77d accessions almost always showed the lowest expression levels among the spelt accessions. The diploid accessions displayed more contrasting results. The TR08 accession showed no fluorescence for the DQ2.5-glia-α1, DQ2.5-glia-α2 and DQ2.5-glia-α3 epitopes. In the same way, the LB01 accession showed no expression for the DQ2.5-glia-α2 epitope and almost none for the DQ8-glia-α1 epitope.

**Fig. 4:**
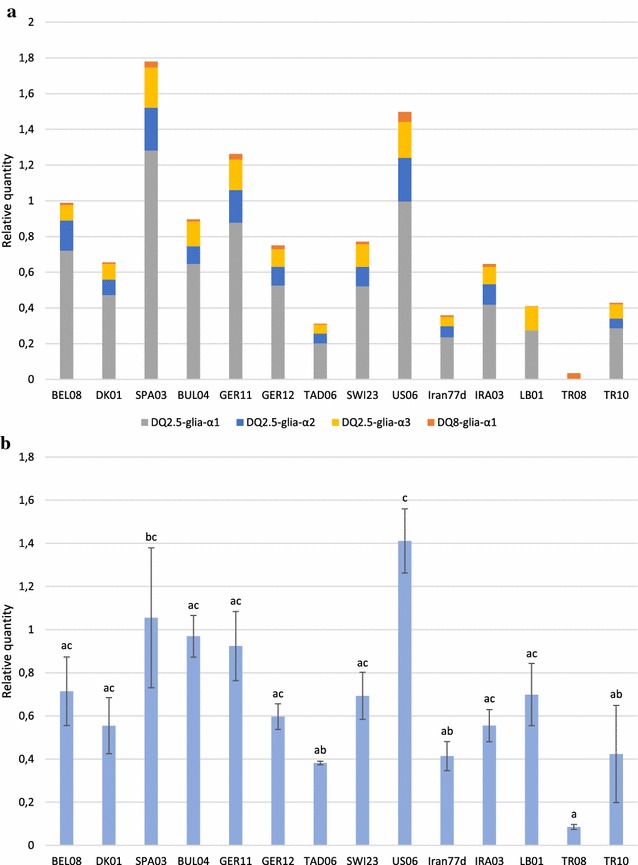
Expression profile of the cumulated canonical epitopes and the global α-gliadins among spelt and diploid accessions. **a** Relative quantification of the expression of the four cumulated canonical α-gliadin epitopes affecting CD patients among the 11 contrasted spelt accessions and the three diploid species *T. urartu*, *Ae. speltoides* and *Ae. tauschii.* The relative quantity of each epitope in the α-gliadin transcripts was calculated by the $2^{{\Delta {\text{C}}_{\text{t}} }}$ method through the normalization of qPCR values to those obtained for the four most stable reference genes and the calibration to a unique α-gliadin clone displaying the four canonical epitopes in one copy. **b** Relative quantification of the expressed α-gliadin sequences in the same accessions. The sample with the lowest C_t_ value was chosen as calibrator. *Error bars* represent the standard error of the mean and the *letters* displayed above denote significant differences highlighted by Tukey’s multiple comparison test

The relative quantity of each canonical epitope was compared thanks to the calibration of qPCR values to a unique α-gliadin clone displaying the four canonical epitopes in one copy (Fig. 4a). Unsurprisingly, the SPA03 and US06 accessions showed the highest global expression values and the TAD06 and Iran77d accessions the lowest ones. The *Ae. speltoides* accession TR08 showed the highest expression for the canonical DQ8-glia-α1 epitope, but the low relative expression of this epitope compared to the others combined with the absence of expressed canonical DQ2.5-glia-α1, -α2 and -α3 epitopes in this accession led to a very low cumulative expression level for this accession. Intermediate comparable expression levels were measured for the DQ2.5-glia-α2 and DQ2.5-glia-α3 epitopes, whereas the DQ2.5-glia-α1 epitope clearly showed the highest expression compared to the three other epitopes.

### Alpha-gliadin expression analysis

With the aim of investigating to what extent the measured relative quantities of canonical epitopes are related to the global α-gliadin expression level, an analysis was carried out using the SYBR^®^ dye to detect α-gliadin amplicons, whatever the epitope variants (Fig. 4b). Again, the analysis of variance revealed statistically significant differences in the α-gliadin expression levels from one accession to another (ANOVA F_13_ = 5.1367; *P* = 2.256e−3). The diploid species *Ae. speltoides* (TR08) and *Ae. tauschii* (TR10) showed the lowest α-gliadin expression levels whereas a much higher value was reached for the remaining diploid species, *T. urartu* (LB01). In spelt accessions, the American US06 and the Iranian Iran77d accessions were identified as having the highest and the lowest expression levels, respectively. Interestingly, the amount of expressed canonical epitopes did not always correspond to the global expression level of α-gliadin sequences.

## Discussion

### Development of epitope-targeting TaqMan probes

The objective of this study was to develop a tool to detect and accurately quantify the expression of the four α-gliadin toxic epitopes. The developed tool had to discriminate canonical epitopes from their allelic variants because, even if some natural variants can cause a T-cell response in some patients, only the canonical epitopes display a full toxicity [27]. It was therefore decided to develop TaqMan probes given their ability to discriminate among allelic variants differing by only one SNP.

Four TaqMan probes targeting the four α-gliadin canonical epitopes were developed and their specificity was demonstrated with previously cloned α-gliadin sequences. This specificity was confirmed by testing the probes with three diploid accessions (*T. urartu*, *Ae. speltoides* and *Ae. tauschii*), which are representative of the three ancestral genomes of spelt and bread wheat: the A, B and D genomes respectively. No fluorescent signal was noted for *Ae. speltoides* with the DQ2.5-glia-α1-, DQ2.5-glia-α2- and DQ2.5-glia-α3-targeting probes, and a very low expression level was measured for *T. urartu* with the DQ8-glia-α1-targeting probe (Fig. 3). This is consistent with the previously demonstrated absence of canonical DQ2.5-glia-α1, DQ2.5-glia-α2 and DQ2.5-glia-α3 epitopes in B genome α-gliadins [16, 27, 43] and the very low proportion of canonical DQ8-glia-α1 epitope in A genome α-gliadins [44].

The TaqMan probe’s specificity was tested with α-gliadin clones, ensuring that the probe does not hybridize to any known allelic variant of the epitopes found in spelt sequences. This specificity makes TaqMan probes a very accurate tool to infer the expression of the different toxic epitopes.

Mitea et al. [27] studied the epitope composition of 3022 expressed α-gliadin sequences originating from 11 bread wheat varieties to analyze the toxicity of each variant of the four epitopes. Almost every allelic variant found in bread wheat sequences was also identified in spelt sequences [16]. Moreover, the SNPs displayed in the remaining allelic variants in bread wheat sequences are located in the hybridization site of the probes developed in the present study. This makes the TaqMan probes designed in this project useful to study the immunogenic potential of both spelt and bread wheat α-gliadin sequences.

### Selection of stable reference genes and development of specific TaqMan probes

The quantification of the gene expression level through qPCR requires a normalization step in order to accurately compare samples with one another. Reference genes showing a stable expression are usually used for this purpose. The normalization of qPCR data obtained with TaqMan probes thus required the use of additional TaqMan probes specific to such reference genes. Paolacci et al. [41] studied the expression stability of 32 reference genes using the cDNAs from 24 bread wheat plant samples, including different tissues, developmental stages and temperature stresses. They developed specific primers and used them in qPCR amplifications to assess the expression stability of the reference genes. We selected the most stable reference genes in developing grains on the basis of their results and designed specific primers/TaqMan probes systems to fit with the TaqMan amplification requirements. TaqMan probes specific to six reference genes were developed in this way and the most stable genes as well as the appropriate number of genes to use for normalization were determined. These reliable probes represent a normalization tool that could be useful in other studies where the expression level of sequences other than α-gliadins is analyzed.

### Epitope expression profile in spelt accessions

The qPCR values measured with the probes focusing on the four canonical epitopes showed very different expression patterns according to the epitope (Fig. 4a). The canonical DQ2.5-glia-α1 epitope is by far the most strongly expressed, followed by DQ2.5-glia-α2, DQ2.5-glia-α3 and finally DQ8-glia-α1 to a lesser extent. DQ2.5-glia-α1 and DQ2.5-glia-α2 are overlapping epitopes which are displayed in one, two or three copies in the D genome. Three copies of each epitope lead to the full 33-mer fragment, which is the most immunogenic fragment of α-gliadin sequences [25, 26]. Moreover, the DQ2.5-glia-α1 epitope is encountered in its canonical form in the D but also in the A genome [43]. Combined with its possible duplication or triplication, this explains why the highest expression is observed for this epitope.

The analysis of epitope expression levels in different accessions also highlighted high variations. It has been shown by a sequencing analysis in a previous study [16] that spelt α-gliadins from the A genome were expressed most strongly and could display three out of four epitopes in their canonical form. The same study highlighted that SPA03 and US06 were the two accessions with the highest proportion of A genome α-gliadins. This supports the results obtained in the present study identifying the SPA03 and US06 accessions as those with the highest canonical epitope expression levels. The lowest expression values measured for TAD06 are also consistent with our previous results, in which the greatest proportion of B genome α-gliadins—displaying a high proportion of epitope variants—were found in this Tajik accession.

Unsurprisingly, the amount of expressed canonical epitopes is related to the expression level of α-gliadin transcripts. However, the global amount of expressed α-gliadins does not provide enough information to study the immunogenic content of the accessions, since some opposed trends can be identified between Fig. 4a, b: the quantification of the global α-gliadin expression level can lead to an under- or an over-estimation of the canonical epitope expression level, as noticed for the SPA03 and BUL04 or LB01 accessions, respectively. Generally speaking, although a correlation might appear for some accessions between the toxic epitope detection and the global α-gliadin expression levels, measures with epitope-targeting probes provide more precise information and demonstrates the usefulness of this TaqMan probes for developing new varieties with reduced immunogenic content without lowering the overall amount of expressed α-gliadins.

Interestingly, the three Asian spelt accessions included in the present study displayed the lowest canonical epitope expression levels, compared to the seven European accessions and the American one—presumably of European origin [14]. Several authors have suggested the existence of two independent origins for spelt—one in Asia and one in Europe—and genetic differences have been reported [45–48]. Asian spelt seems to have emerged, like bread wheat, through hybridization between the cultivated free-threshing tetraploid wheat *Triticum turgidum* ssp. *turgidum* (AABB genome) and the wild *Ae. tauschii* (DD genome), whereas European spelt may be the result of a secondary hybridization between bread wheat and cultivated non-free-threshing tetraploid emmer [*Triticum turgidum* ssp. *diccocum* (Schrank ex Schübler) Thell., AABB genome]. The lower canonical epitope expression levels measured in Asian spelt accessions could be linked to their putative distinct origin from European spelts. This opens up an interesting route to explore the α-gliadin immunogenic content of Asian spelt accessions with the aim of developing safer varieties for CD patients.

### Comparison between the developed TaqMan probe method and other existing techniques

The developed probes involve the extraction of mRNA from immature seeds which requires a careful handling. However, this methodology enables to quantify the expression of the four major T-cell stimulatory epitopes related to CD independently on the same sample. This tool has been developed to carry out genetic studies of the toxic potential found in α-gliadin transcripts from spelt or bread wheat accessions, with the aim of applying them in breeding programs. It could be useful to track modifications at the sequence or at the expression level in molecular breeding. Conversely, ELISA kits focus only on one or two CD-related epitopes but are not completely specific to canonical epitopes. They are thus mostly intended to measure the global amount of gluten in food samples rather than to quantify the toxic content held in these samples. Beside ELISA, the methodology based on aptamers—binding the immunodominant 33-mer peptide—developed by Amaya-Gonzalez et al. [36] enables to quantify the gluten content through an electrochemical competitive enzyme-linked assay on magnetic particles. The selected aptamer is six times more sensitive than the reference ELISA test and it does not show any cross-reactivity with non-toxic proteins such as those found in maize, soya and rice. Nevertheless, these aptamers do not focus on individual epitopes and, despite their high sensitivity, the absence of cross-reactivity with allelic variants of the 33-mer has not been demonstrated.

In the recent years, another strategy coupling liquid chromatography (LC) to mass spectrometry (MS) has been developed [34, 35]. Like TaqMan probes developed in this work, the LC-MS technique enables to discriminate canonical epitopes from their allelic variants.

ELISA tests, aptamer assays and LC-MS are intended to detect gluten at the protein level, whereas TaqMan probes focus on α-gliadin transcripts. Given that the component responsible for triggering the disease is the protein, the toxic content of a flour cannot be irrevocably stated from analyses carried out on mRNA samples since slight variations can still occur between the mRNA and the protein stages. However, Van den Broeck et al. [35] showed that their quantification results, based on LC-MS method, were correlated with those of Salentijn et al. [49], who developed an RNA sequencing pipeline to analyze which α-gliadin sequences are being expressed.

The identification or the development of spelt or bread wheat accessions with a reduced immunogenic content is not sufficient for celiac disease patients since the low amount of toxic epitopes will still trigger the disease. However, the developed TaqMan probes could be used in conventional and/or molecular breeding programs (genome editing,…) aiming at developing celiac-safe varieties by tracking the immunogenic potential at the genetic level. This longer-term application of the designed probes could thus find an interest for the development of safe varieties for CD patients.

In addition to the application of the developed probes on α-gliadin transcripts, they could be used as a first approximation on the genomic DNA (gDNA) of spelt and bread wheat accessions if the results obtained from gDNA samples are globally correlated with those obtained from cDNA samples.

## Conclusions

In this study, we have developed a tool to study the expression level of the four major α-gliadin T-cell stimulatory epitopes involved in celiac disease. This was achieved by designing, first, TaqMan probes that only hybridize to the canonical form of each epitope, and, second, TaqMan probes targeting stable reference genes. This work thus provides reliable tools to study the expression of the four α-gliadin canonical epitopes as well as for normalization purposes in studies where the expression level of sequences other than α-gliadins is analyzed. The application of the designed probes to contrasted spelt accessions revealed high variations in the expression levels of the canonical epitopes. In particular, 11 spelt accessions were analyzed and the three Asian spelts showed a low expression of these epitopes compared to the eight European ones. Even if more accessions are needed to draw definitive conclusions, these differing results between European and Asian spelts seem to be a way worth exploring, since it could open up an interesting route in the development of cereal varieties which are safe for CD patients.

## Additional files

**Additional file 1.** Composition of the canonical form and the allelic variants of the four α-gliadin T-cell stimulatory epitopes used to optimize each TaqMan probe’s specificity. The file presents the canonical form and the allelic variants of the four α-gliadin T-cell stimulatory epitopes on which the specificity of the developed epitope-targeting probes was tested. The GeneBank accession numbers refer to α-gliadin sequences displaying the different epitope variants used to study the probe specificity.

**Additional file 2.** Melting curve analyses carried out after measurement by qPCR of the global amount of expressed α-gliadin sequences in 11 spelt (BEL08, DK01, SPA03, BUL04, GER11, GER12, TAD06, SWI23, US06, Iran77d and IRA03) and three diploid (LB01, TR08 and TR10) accessions representative of the ancestral genomes of spelt and bread wheat. The file presents the melting curve analyses performed after the amplification of α-gliadin sequences with SYBR® dye to check that only one amplicon has been amplified in each sample.

**Additional file 3.** Primer efficiency calculation for the four T-cell stimulatory epitopes involved in CD and for the four reference genes used to normalize the epitope expression levels. The file presents the efficiency calculation results obtained for the four most stable reference genes used in this study to normalize epitope expression levels.

**Additional file 4.** Agarose gel electrophoresis of the qPCR products amplified with primers targeting the four most stable reference genes ARF, RLI, VAS and DUF52. The file presents the amplification profile obtained after running on a 2% agarose gel the qPCR products amplified from spelt cDNA with the primers focusing on the reference genes ARF, RLI, VAS and DUF52.

**Additional file 5.** Average Ct values measured for the four T-cell stimulatory epitopes involved in CD and for the four most stable references genes in 11 spelt accessions and 3 diploid accessions representative of the ancestral genomes of spelt and bread wheat. The file presents the mean Ct values measured with TaqMan probes focusing on the four CD-related epitopes and the four reference genes, which were used to calculate the epitope expression levels in each sample.
