# Supplementary material for: Development of TaqMan probes targeting the four major celiac disease epitopes found in α-gliadin sequences of spelt (Triticum aestivum ssp. spelta) and bread wheat (Triticum aestivum ssp. aestivum)
Source: Plant Methods. 2017 Sep 6;13:72. doi: 10.1186/s13007-017-0222-2 (PMC5588674; doi:10.1186/s13007-017-0222-2)
Supplement: Supplementary file 1 — Additional file 1. Composition of the canonical form and the allelic variants of the four α-gliadin T-cell stimulatory epitopes used to optimize each TaqMan probe’s specificity. The file presents the canonical form and the allelic variants of the four α-gliadin T-cell stimulatory epitopes on which the specificity of the developed epitope-targeting probes was tested. The GeneBank accession numbers refer to α-gliadin sequences displaying the different epitope variants used to study the probe specificity. [file 13007_2017_222_MOESM1_ESM.pdf]

Additional file 1. Composition of the canonical form and the allelic variants of the four α-gliadin T-cell stimulatory epitopes used to optimize each TaqMan probe's specificity.

Epitopes	Variants		Composition [1]	GeneBank accession numbers [2]
DQ2.5-glia-α1	Canonical epitope	NA	CCA T{T/A}T CC{G/A} CAA CTA CCA TAT	KX173976, KX174272, KX174055
		AA	P{F/Y}PQPQLPY	
	Mutated epitopes	NA	CCA TTT CCG --- CCA CAA CTA CCA TAT	KX173988
		AA	PFP-PQLPY	
		NA	CCA TTT TCG CAG CCG CAA CTA CCA TAT	KX173847
		AA	PFSQPQLPY	
		NA	CCA TTT CTG CAG CCG CAA CTA CCA TAT	KX174174
		AA	PFLQPQLPY	
		NA	CCA TTT CCG CAG CCG CAA CTA TCA TAT	KX174120
		AA	PFPQPQLSY	
		NA	CCA TAT CCG CGG CCG CAA CTA CCA TAT	KX174253
		AA	PYPRPQLPY	
		NA	CCA TAT CCG CAG CCG CAT CTA CCA TAT	KX174252
		AA	PYPQPHLPY	
DQ2.5-glia-α2	Canonical epitope	NA	CC{A/G} CAG CCG CAA CTA CCA TAT CCG CAG	KX173976, KX174272, KX174055
		AA	PQPQLPYPQ	
	Mutated epitopes	NA	CCG --- CCA CAA CTA CCA TAT CCG CAG	KX173988
		AA	P-PQLPYPQ	
		NA	CCG CAG CCG CAA CTA CCA TAT TCA CAG	KX174127
		AA	PQPQLPYSQ	
		NA	CCG CGG CCG CAA CTA CCA TAT CCG CAG	KX174253
		AA	PRPQLPYPQ	
		NA	CCG CAG CCG CAT CTA CCA TAT CCG CAG	KX174252
		AA	PQPHLPYPQ	
DQ2.5-glia-α3	Canonical epitope	NA	TTT CGA CCA CAA CAA CCA TAT CCA CA{A/G}	KX174127
		AA	FRPQQPYPQ	
	Mutated epitopes	NA	TTT CCA CCA CAA CAA CCA TAT CCA CAA	KX173988
		AA	FPPQQPYPQ	
		NA	TTT CGA CAA CAA CAA CCA TAT CCA CAA	KX173847
		AA	FRQQQPYPQ	
		NA	TTT CGA CCA CAA CAA TCA TAT CCA CAA	KX174255
		AA	FRPQQSYPQ	
		NA	TTT CGA CCA CAA AAA CCA TAT CCA CAA	KX173906
		AA	FRPQKPYPQ	
		NA	TTT CAA CCA CAA CAA CCA TAT CCA CAA	KX174012
		AA	FQPQQPYPQ	
DQ8-glia-α1	Canonical epitope	NA	CAG GGC TCC TTC CAG CCA TCT CAG CAA	KX174252
		AA	QGSFQPSQQ	
	Mutated epitopes	NA	CAG GGC TCC TTC CGG CCA TCT CAG CAA	KX173906
		AA	QGSFRPSQQ	
		NA	CAG GGC TCC TTC CAG TCA TCT CAG CAA	KX173989
		AA	QGSFQSSQQ	
		NA	CAG GGC TTC TTC CAG CCA TCT CAG CAA	KX173976
		AA	QGFFQPSQQ	
		NA	CAG GTC TCC TTC CAG CCA TCT CAG CTA	KX173988
		AA	QVSFQPSQL	

[1] : NA : Nucleic acid composition ; AA : Amino acid composition

[2] : α-gliadin sequences previously cloned in Dubois et al. 2016
