# Supplementary material for: Development of TaqMan probes targeting the four major celiac disease epitopes found in α-gliadin sequences of spelt (Triticum aestivum ssp. spelta) and bread wheat (Triticum aestivum ssp. aestivum)
Source: Plant Methods. 2017 Sep 6;13:72. doi: 10.1186/s13007-017-0222-2 (PMC5588674; doi:10.1186/s13007-017-0222-2)
Supplement: Supplementary file 2 — Additional file 2. Melting curve analyses carried out after measurement by qPCR of the global amount of expressed α-gliadin sequences in 11 spelt (BEL08, DK01, SPA03, BUL04, GER11, GER12, TAD06, SWI23, US06, Iran77d and IRA03) and three diploid (LB01, TR08 and TR10) accessions representative of the ancestral genomes of spelt and bread wheat. The file presents the melting curve analyses performed after the amplification of α-gliadin sequences with SYBR® dye to check that only one amplicon has been amplified in each sample. [file 13007_2017_222_MOESM2_ESM.pdf]

Additional file 2. Melting curve analyses carried out after measurement by qPCR of the global amount of expressed α-gliadin sequences in 11 spelt (BEL08, DK01, SPA03, BUL04, GER11, GER12, TAD06, SWI23, US06, Iran77d and IRA03) and three diploid (LB01, TR08 and TR10) accessions representative of the ancestral genomes of spelt and bread wheat.

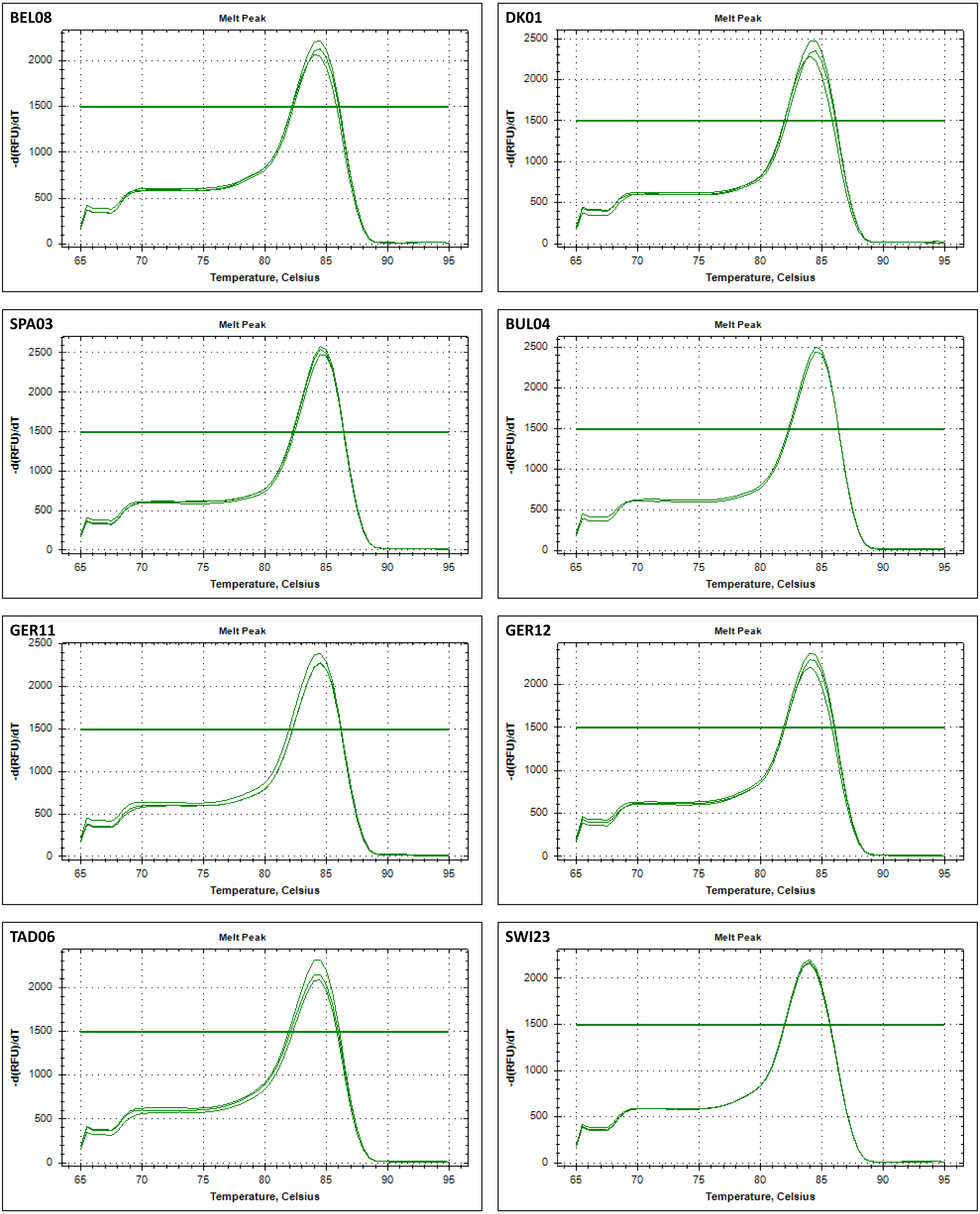

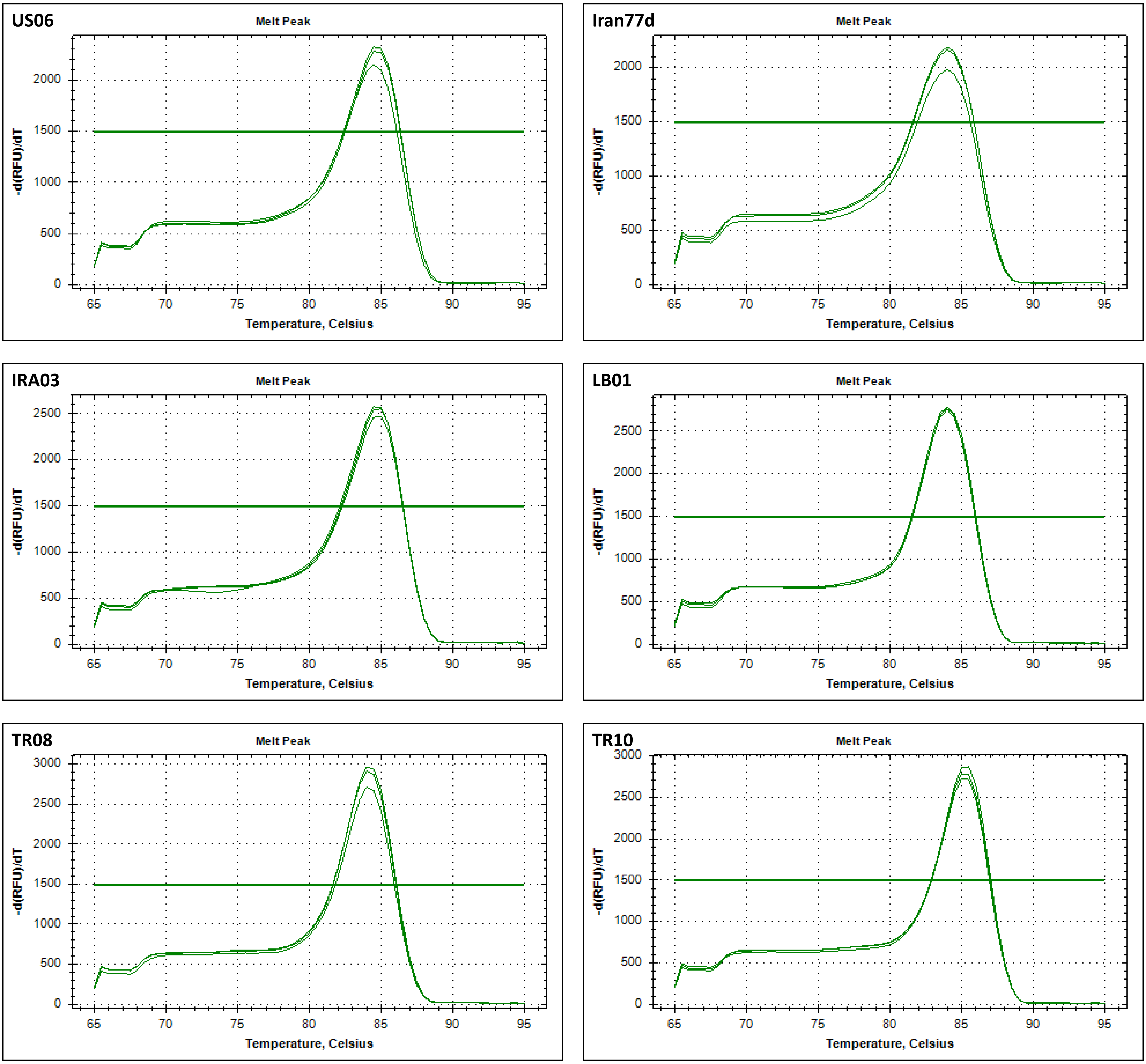
US06
Melt Peak
-d(RFU)/dT
Temperature, Celsius
Iran77d
Melt Peak
-d(RFU)/dT
Temperature, Celsius
IRA03
Melt Peak
-d(RFU)/dT
Temperature, Celsius
LB01
Melt Peak
-d(RFU)/dT
Temperature, Celsius
TR08
Melt Peak
-d(RFU)/dT
Temperature, Celsius
TR10
Melt Peak
-d(RFU)/dT
Temperature, Celsius
