# Supplementary material for: Development of TaqMan probes targeting the four major celiac disease epitopes found in α-gliadin sequences of spelt (Triticum aestivum ssp. spelta) and bread wheat (Triticum aestivum ssp. aestivum)
Source: Plant Methods. 2017 Sep 6;13:72. doi: 10.1186/s13007-017-0222-2 (PMC5588674; doi:10.1186/s13007-017-0222-2)
Supplement: Supplementary file 3 — Additional file 3. Primer efficiency calculation for the four T-cell stimulatory epitopes involved in CD and for the four reference genes used to normalize the epitope expression levels. The file presents the efficiency calculation results obtained for the four most stable reference genes used in this study to normalize epitope expression levels. [file 13007_2017_222_MOESM3_ESM.pdf]

Additional file 3. Primer efficiency calculation for the four T-cell stimulatory epitopes involved in CD and for the four reference genes used to normalize the epitope expression levels.

	Targeted sequence	Slope	R^2	Efficiency (%)
Epitopes	DQ2.5-glia-α1	-3.382	0.999	97.5
	DQ2.5-glia-α2	-3.350	0.999	98.8
	DQ2.5-glia-α3	-3.333	0.999	99.5
	DQ8-glia-α1	-3.412	0.997	96.4
Reference genes [1]	ARF	-3.344	0.994	99.1
	RLI	-3.395	0.998	97.0
	VAS	-3.322	0.998	100.0
	DUF52	-3.347	0.994	99.0

Probes targeting canonical epitopes:

1. DQ2.5-glia-α1

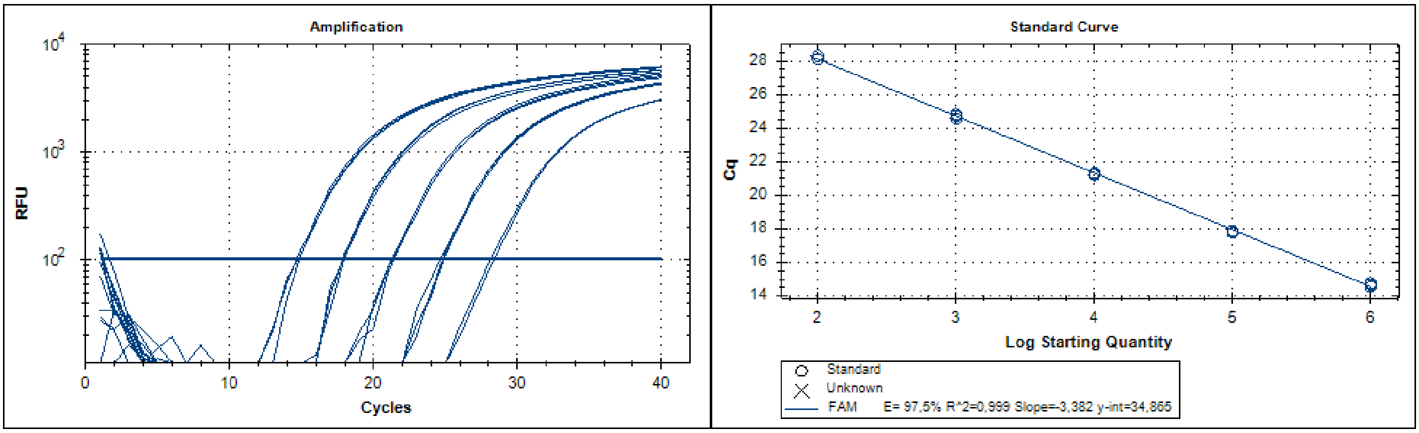

2. DQ2.5-glia-α2

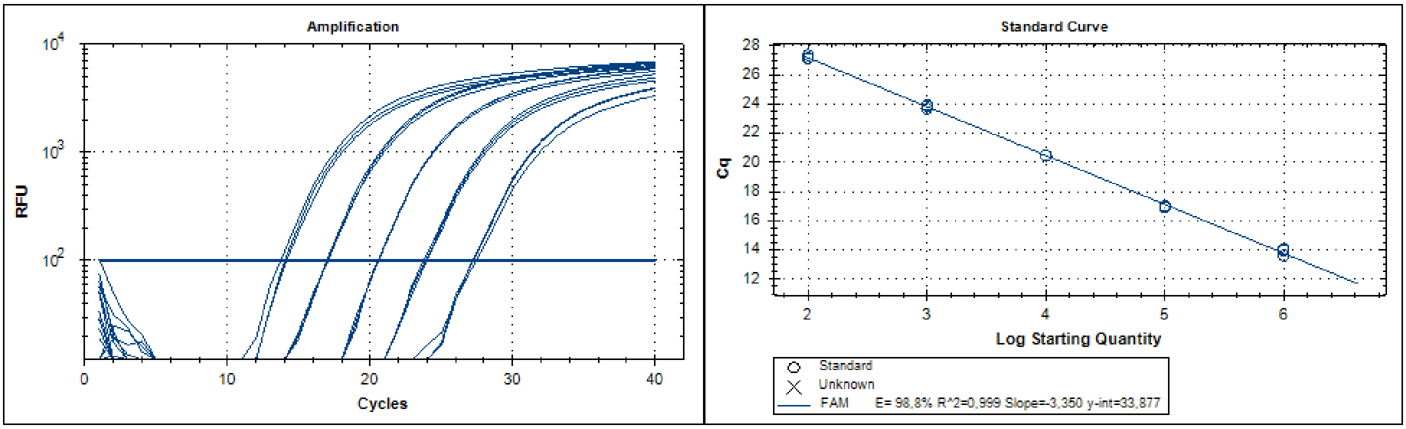

3. DQ2.5-glia-α3

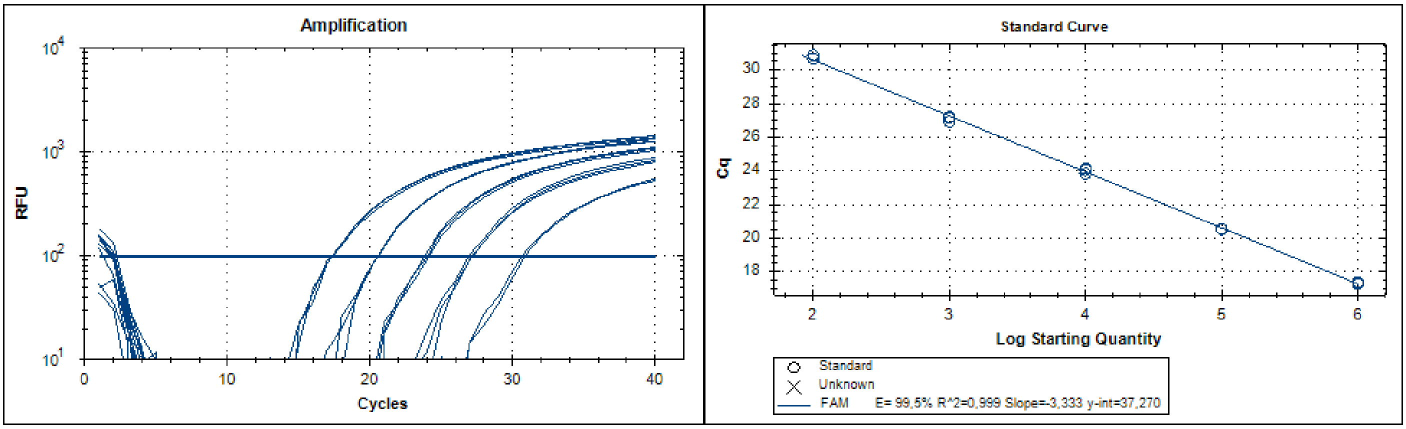

4. DQ8-glia-α1

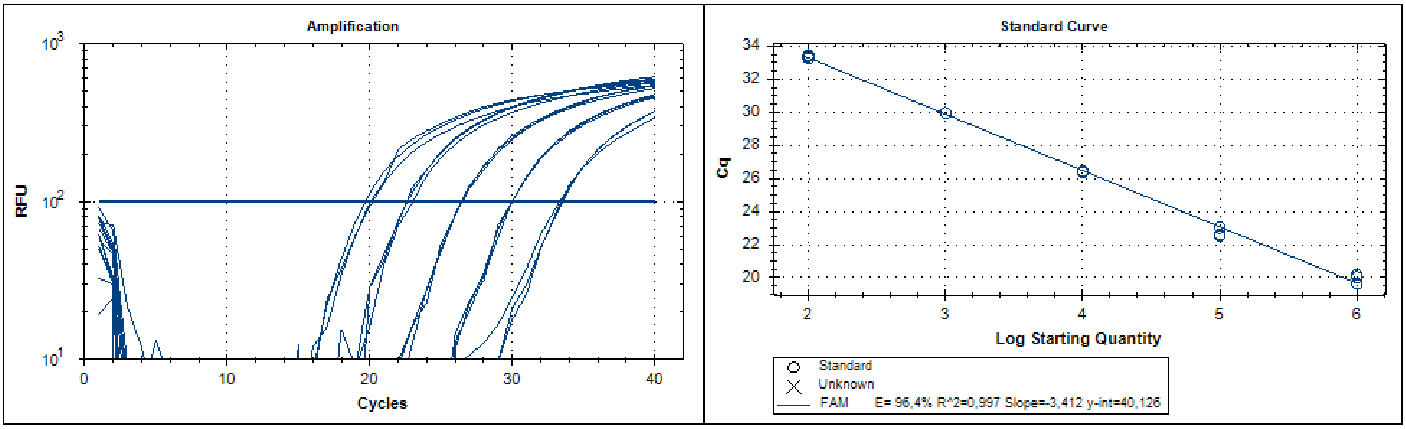

Probes targeting reference genes:

5. ARF

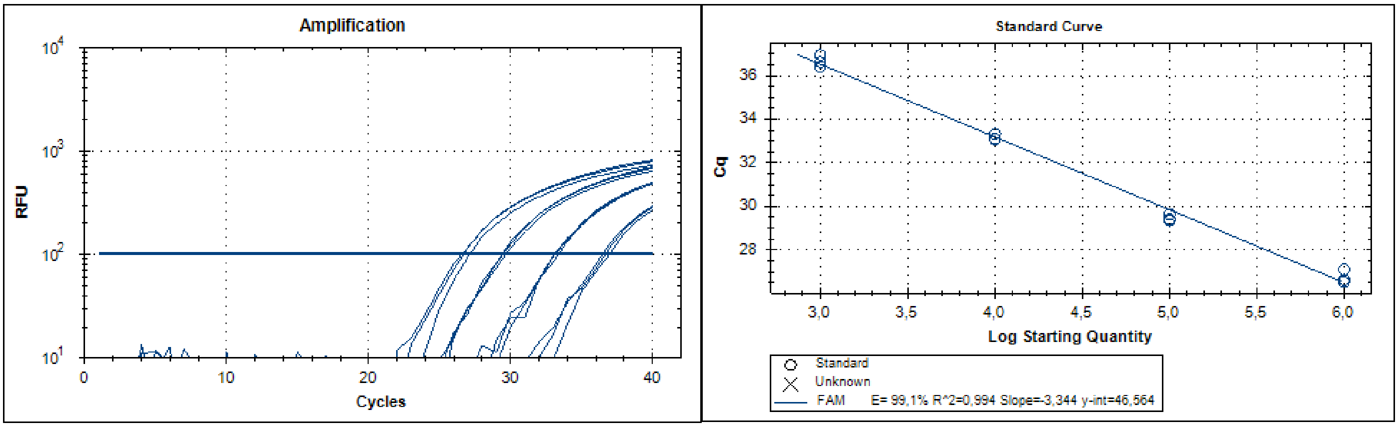

6. RLI

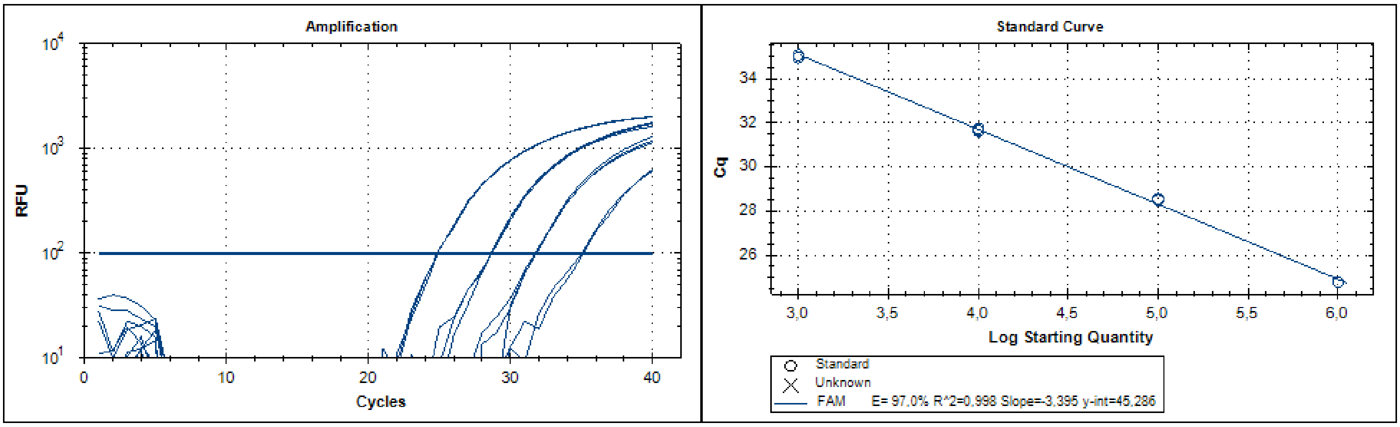

7. VAS

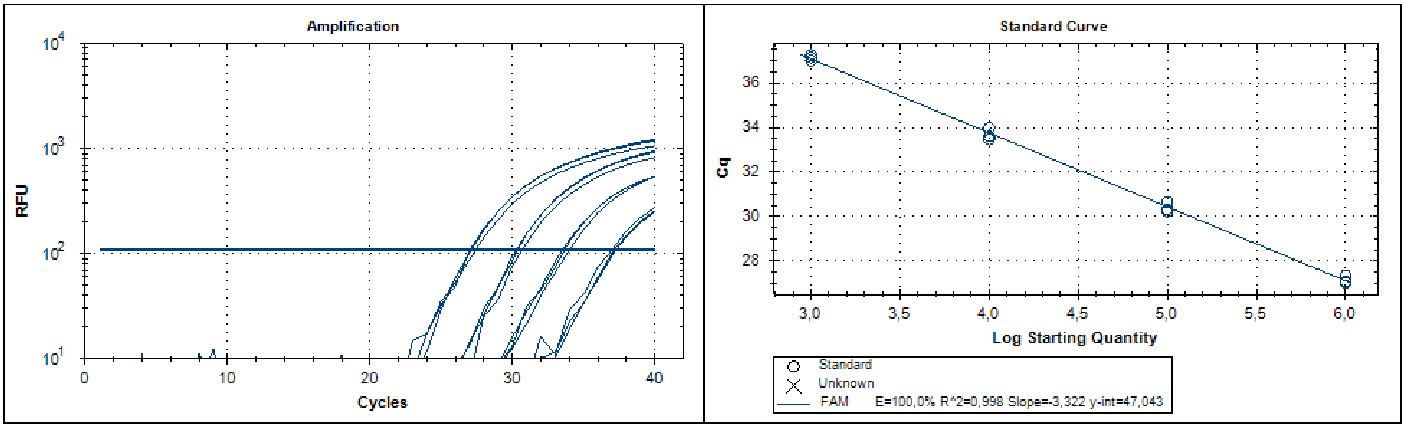

8. DUF52

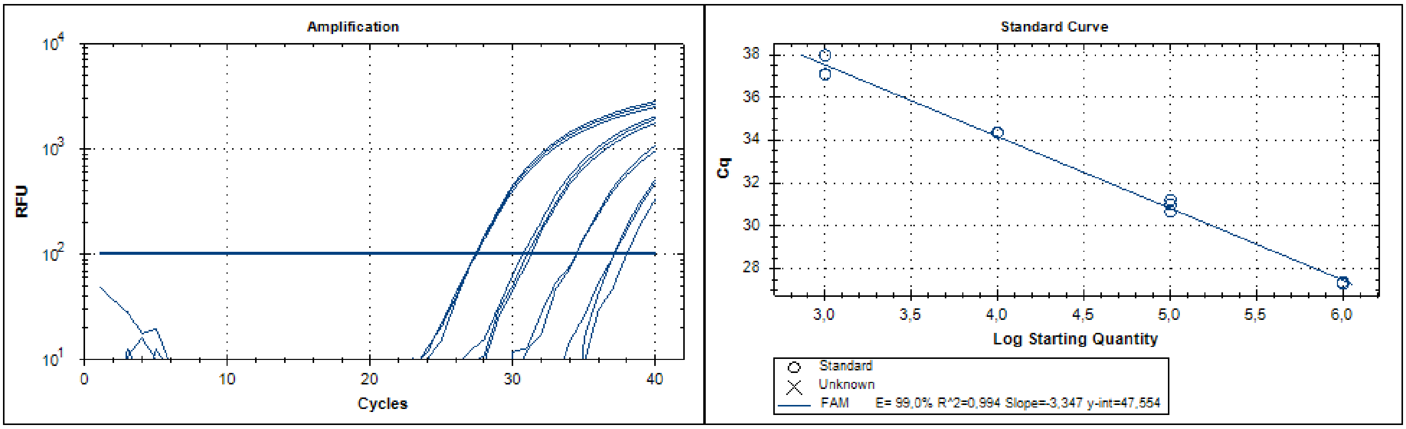

Each set of primers was validated for its efficiency by carrying out qPCR amplifications with successive 10-fold cDNA dilutions. Calibration curves were drawn by plotting the logarithm of the cDNA concentration on the x-axis and the corresponding C_t values on the y-axis. The efficiency was then calculated based on the curve slope: Efficiency (%) = $[(10^{-1/slope})-1] * 100$.

[1] : ARF: ADP-ribosylation factor, RLI: Similar to RNase L inhibitor-like protein, VAS: Vacuolar ATP synthase 16 kDa proteolipid sub., DUF52: Protein of unknown function [DUF52 family].
