# Supplementary material for: Development of TaqMan probes targeting the four major celiac disease epitopes found in α-gliadin sequences of spelt (Triticum aestivum ssp. spelta) and bread wheat (Triticum aestivum ssp. aestivum)
Source: Plant Methods. 2017 Sep 6;13:72. doi: 10.1186/s13007-017-0222-2 (PMC5588674; doi:10.1186/s13007-017-0222-2)
Supplement: Supplementary file 4 — Additional file 4. Agarose gel electrophoresis of the qPCR products amplified with primers targeting the four most stable reference genes ARF, RLI, VAS and DUF52. The file presents the amplification profile obtained after running on a 2% agarose gel the qPCR products amplified from spelt cDNA with the primers focusing on the reference genes ARF, RLI, VAS and DUF52. [file 13007_2017_222_MOESM4_ESM.pdf]

Additional file 4. Agarose gel electrophoresis of the qPCR products amplified with primers targeting the four most stable reference genes ARF, RLI, VAS and DUF52.

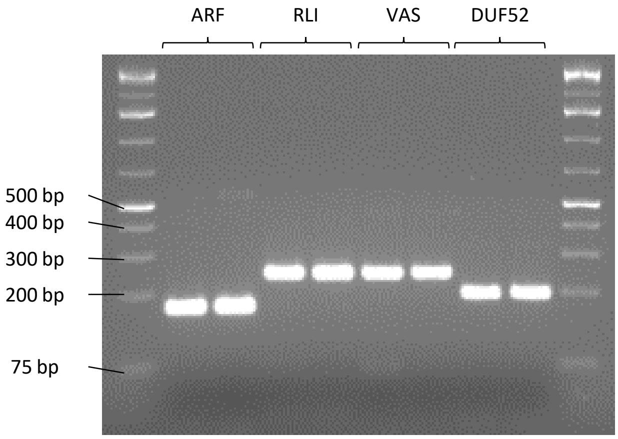

ARF: ADP-ribosylation factor, RLI: Similar to RNase L inhibitor-like protein, VAS: Vacuolar ATP synthase 16 kDa proteolipid sub., DUF52: Protein of unknown function [DUF52 family].
