# Supplementary material for: Development of TaqMan probes targeting the four major celiac disease epitopes found in α-gliadin sequences of spelt (Triticum aestivum ssp. spelta) and bread wheat (Triticum aestivum ssp. aestivum)
Source: Plant Methods. 2017 Sep 6;13:72. doi: 10.1186/s13007-017-0222-2 (PMC5588674; doi:10.1186/s13007-017-0222-2)
Supplement: Supplementary file 5 — Additional file 5. Average Ct values measured for the four T-cell stimulatory epitopes involved in CD and for the four most stable references genes in 11 spelt accessions and 3 diploid accessions representative of the ancestral genomes of spelt and bread wheat. The file presents the mean Ct values measured with TaqMan probes focusing on the four CD-related epitopes and the four reference genes, which were used to calculate the epitope expression levels in each sample. [file 13007_2017_222_MOESM5_ESM.pdf]

Additional file 5. Average Ct values measured for the four T-cell stimulatory epitopes involved in CD and for the four most stable references genes in 11 spelt accessions and 3 diploid accessions representative of the ancestral genomes of spelt and bread wheat.

Species	Accession name	TaqMan probes targeting epitopes				TaqMan probes targeting reference genes			
		A20	A1	A9	A2	ARF	RLI	VAS	DUF52
Triticum aestivum ssp. *spelta*	BEL08	19.62	21.90	16.59	15.15	27.64	29.92	32.03	29.36
	DK01	18.88	21.80	16.44	15.37	26.70	29.55	31.49	28.60
	SPA03	18.30	20.47	15.80	14.68	28.01	29.89	31.32	30.16
	BUL04	21.05	24.14	18.76	17.99	29.27	32.54	34.32	31.36
	GER11	18.83	20.75	16.43	15.18	27.06	30.39	32.49	29.52
	GER12	19.60	21.19	17.08	15.91	27.82	30.34	31.96	29.61
	TAD06	19.85	22.67	17.77	16.16	27.27	29.68	31.10	28.86
	SWI23	18.68	21.23	16.58	15.32	27.25	29.76	31.66	28.94
	US06	19.74	18.91	17.37	15.91	29.00	31.58	33.21	30.77
	Iran77d	21.03	22.79	18.72	17.11	28.59	30.66	32.02	30.19
	IRA03	19.19	21.28	17.03	15.38	27.36	30.14	31.58	29.03
Triticum urartu	LB01	19.97	35.08	18.83		27.90	32.00	33.58	29.35
Aegilops speltoides	TR08		22.09			28.07	31.41	34.68	31.83
Aegilops tauschii	TR10	22.61	24.97	20.53	19.40	29.73	33.45	34.75	31.25
